# Supplementary material for: Hydrogel-Based Pre-Clinical Evaluation of Repurposed FDA-Approved Drugs for AML
Source: Int J Mol Sci. 2023 Feb 20;24(4):4235. doi: 10.3390/ijms24044235 (PMC9966469; doi:10.3390/ijms24044235)
Supplement: Supplementary file 1 [file ijms-24-04235-s001.zip › ijms-2187765-supplementary.pdf]

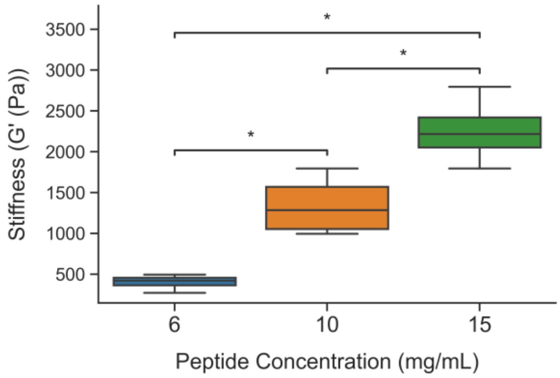

Figure S1A. Stiffness (G′) of peptide hydrogels of varying concentrations, assessed via bulk oscillatory rheology. Results demonstrate a positive relationship between peptide concentration and stiffness (G′), and a significant difference in stiffness between peptide concentrations tested. (Independent t-test; n=4; *: $p < 0.05$; **: $p < 0.01$; ***: $p < 0.001$). The average stiffness of 6 mg/mL peptide hydrogels was 402 Pa. This is within the same range of stiffness previously reported for Matrigel® (400 - 420 Pa). Therefore, 6 mg/mL was selected as the concentration for this study.

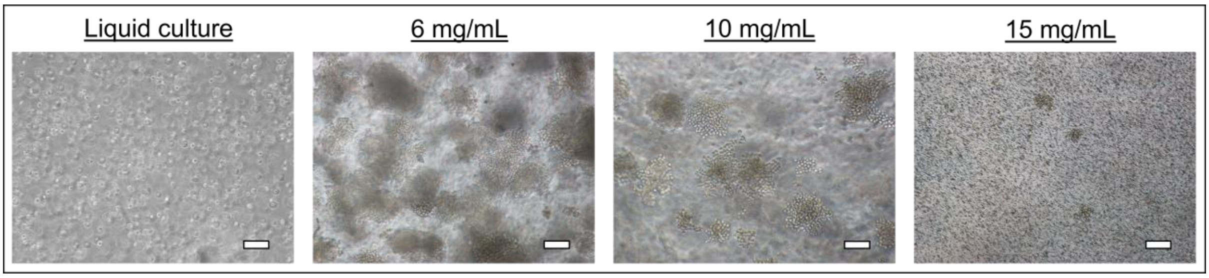

Figure S1B. Representative phase contrast images of U937 cell growth in increasing concentration of peptide. Images taken 7 days after initial seeding. Scale bars = 100 μm

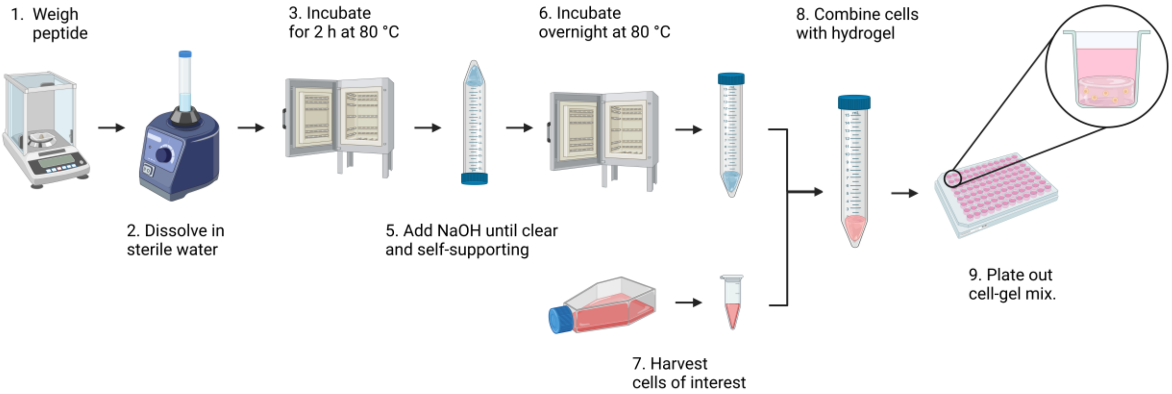

Figure S2 – Schematic overview of the peptide hydrogel preparation and cell encapsulation process.

Table S1. AML patient sample diagnostic data

Sample ID	Age (years)	Sex	FLT3 Status	NPM1 Status
PB522	64	Female	Wild type	Wild type
MJ694	69	Male	Wild Type	Mutant
